# Aerobic capacity and future cardiovascular risk in Indian community from a low-income area in Cauca, Colombia

**DOI:** 10.1186/s13052-017-0347-y

**Published:** 2017-03-07

**Authors:** Robinson Ramírez-Vélez, Jorge Enrique Correa-Bautista, Jeison Alexander Ramos-Sepúlveda, Carlos Andrés Piñeros-Álvarez, Lorena Isabel Giraldo, Mikel Izquierdo, Antonio García-Hermoso, Fernando Rodríguez-Rodríguez, Carlos Cristi-Montero

**Affiliations:** 1 Centro de Estudios para la Medición de la Actividad Física «CEMA», Escuela de Medicina y Ciencias de la Salud, Universidad del Rosario, Bogota, DC 111221 Colombia; 2 Grupo de Investigación en Pedagogía, Licenciatura en Ciencias del Deporte y la Educación Física, Institución Universitaria Antonio José Camacho, Cali, Colombia; 3 Department of Health Sciences, Public University of Navarra, Pamplona, Navarra Spain; 4 Laboratorio de Ciencias de la Actividad Física, el Deporte y la Salud, Facultad de Ciencias Médicas, Universidad de Santiago de Chile, USACH, Santiago, Chile; 5 IRyS Group, Physical Education School, Pontificia Universidad Católica de Valparaiso, Valparaíso, Chile

**Keywords:** Physical fitness, Aerobic fitness, Cardiorespiratory fitness, Risk factors, Chronic diseases, Cardiometabolic health, Indigenous

## Abstract

**Background:**

Several studies indicates that children’s aerobic capacity levels are predictors of the future risk of non-communicable diseases. Therefore, the aim of this study was to establish the proportion of subjects whose aerobic capacity is indicative of future cardiovascular risk in Indian-Nasa community from a low-income area in Cauca, Colombia.

**Methods:**

We performed a cross-sectional analysis of morphological component (height, weight, body mass index (BMI), waist circumference, triceps skinfold, subscapular skinfold, and body fat percent [BF%]), and the cardiorespiratory component (*course-navette* 20 m, shuttle run test and estimation of maximal oxygen consumption by indirect VO_2_max) from 576 participants (319 boys and 257 girls) aged 10 to 17.9 years, using the standardized FUPRECOL test battery.

**Results:**

We showed that the boys performed better than the girls in cardiorespiratory fitness. The proportion of subjects with an aerobic capacity indicative of future cardiovascular risk was 7.3%. By sex, 3.8% of boys and 11.7% of girls (X^2^ *p =* 0.001) displayed an unhealthy aerobic capacity in this study.

**Conclusion:**

The findings of this study that provide the first data on aerobic capacity health for Colombian Nasa Indian children and adolescents aged 10–17.9 years. Although the known loss of aerobic capacity is a serious consequence of the future risk of non-communicable diseases, the deterioration of physical fitness deserves increased attention among indigenous population.

## Background

Health-related fitness consists of cardiorespiratory fitness (CRF), musculoskeletal (e.g., muscle endurance, muscle strength, muscle power), and motor fitness (e.g., balance, coordination and speed of movement), in particular can also be immensely influenced by lifestyle factors [1–6]. Several large cohort studies have shown lack of CRF with high mortality and morbidity is strong, exceeding even that of other classic factors regarding cardiovascular risk factors such as dyslipidaemia, hypertension, or obesity [2–9]. CRF is relates more strongly than physical activity to metabolic and cardiovascular disease risk factors in healthy children and adolescents [3, 6]. Thus, having a high level of CRF in childhood could be a protective factor of cardiovascular disease in adulthood [10].

The effects of cardiovascular risk factors on health may partly be mediated through physical fitness levels. In this context, the inclusion of CRF within health surveillance systems is therefore clearly justifiable, and schools may be an ideal setting for the monitoring of youth fitness. Fitness education and student fitness assessments offer students an opportunity to assess, track, and improve their fitness level. Batteries such as FITNESSGRAM [11], The Presidential Youth Fitness Program (PYFP) [12], The European Physical Fitness (EUROFIT) [13], The Canadian Physical Activity, Fitness and Lifestyle Approach (CPAFLA) [14], and The FUPRECOL battery (in Spanish, *Asociación de la* ***Fu*** *erza* ***Pre*** *nsil con Manifestaciones de Riesgo cardiovascular Tempranas en Niños y Adolescentes* ***Col*** *ombianos*) [15, 16] have been used in diverse studies. The health-related fitness included in the FUPRECOL battery assess the main components of fitness related to health such as (a) morphology and body composition, (b) musculoskeletal fitness, (c) motor fitness, and (d) CRF levels [15].

However, race and ethnicity are also thought to be important determinants of biological and physical determinants of health [17]. Race has traditionally been used to categorize populations on the basis of shared biological characteristics such as genetic variation, skin color, and other observable features [18]. Ethnicity is also traditionally used to categorize a distinctive social and cultural tradition within the group generations [12]. In this context, race and ethnicity is strongly associated with almost every measure of health and disease among indigenous populations including disparities in modifiable risk factors and low participation in physical activity [5, 13–21]. Despite the importance of this, to date there is a lack of scientific evidence regarding the CRF by sex, age, in indigenous populations [21].

In addition to the above, the current state of CRF in Colombia have been poorly documented, particularly among the most vulnerable groups such as rural populations, indigenous, women and children [5]. This will further improve understanding of health-risk levels within CRF categories and help to inform the development of targeted interventions for different race/ethnicity population.

Therefore, the aim of this study was to establish the proportion of subjects whose CRF is indicative of future cardiovascular risk in Indian-Nasa community from a low-income area in Cauca, Colombia.

## Methods

### Participants and sample

We have recently published a complete description of the Colombian Nasa Indian design, methods, and primary outcomes for our current cohort [5]. In brief, the Nasa, Cauca community is located in the Toribio district (South Colombia). Nasa Cauca community is considered a low-income area, with approximately 90% of the 186,178 inhabitants living in the surrounding rural areas [5]. Data from the National Institute of Statistics in Colombia indicate that the estimated prevalence of poverty is substantially higher in the Nasa indigenous (93%) [22].

### Study design

We performed a cross-sectional analysis of baseline data from participants in The FUPRECOL study focused on fitness related to health and non-chronic disease. The recruitment period lasted from June 2014 to January 2015. The analysis was restricted to subjects aged 10–17.9 years, with morphological component, and the cardiorespiratory determinations. The final analytical sample was composed by 576 (319 boys and 257 girls) [5]. A power analysis showed that this sample size was sufficient to estimate the physical fitness with a precision of 11.4% and a power of 80%. The sample size was estimated at 15 to 30 participants per group.

### Morphological measurements

Variables were collected at the same time in the morning, between 7:00 and 10:00 a.m. Body weight and height were measured using standard procedures with electronic scales (Tanita_®_ BC544, Tokyo, Japan) and mechanical stadiometer platform (Seca_®_ 274, Hamburg, Germany), respectively. BMI was calculated as the body weight in kilograms divided by the square of the height in meters. Weight status was defined as having a BMI above the age and sex-specific thresholds of the International Obesity Task Force (IOTF) [23]. Waist circumference (WC) was measured, midway between the lower rib margin and the iliac crest. Skinfold thicknesses (SS) were measured twice on the left side of the body to the nearest 0.1 mm using a Harpenden skinfold caliper (Holtain_®_, Bryberian, UK) at the following sites: (1) triceps SS, halfway between the acromion process and the olecranon process; and (2) subscapular SS, about 20-mm below the tip of the scapula, at an angle of 45° to the lateral side of the body. For both boys and girls percentage body fat (BF%) was calculated using the formulas described previously in children and adolescents by Slaughter et al. [24], Males: 0.735 (triceps + calf) + 1.0; and Females: 0.610 (triceps + calf) + 5.1).

### Cardiorespiratory measurements

The participants performed the international *course-navette* shuttle run test [25]. They ran in a straight line between two lines 20 m apart, while keeping pace with pre-recorded audio signals. The initial speed was 8.5 km/h and was increased by 0.5 km/h per minute [25, 26]. A detailed description of 20-m shuttle run, and to estimate VO_2_max using the formulas described by Leger et al. [25] can be found elsewhere [5].

The FITNESSGRAM standards [27] for age and gender were used to classify the adolescents into those who had reached the Healthy/Unhealthy Fitness Zone or those displaying future cardiovascular risk, which was defined as the minimum level of CRF (in ml*kg*min^−1^ units of VO_2_max) that provides protection against health risks associated with inadequate fitness. Therefore, the participants were classified as having a healthy CRF if their VO_2_max was 40–44 ml*kg*min^−1^ for boys and 38–40 ml*kg*min^−1^ for girls, according to their age. These age-and-sex-specific VO_2_max cut-off points were validated against the presence of metabolic disorders using representative U.S. data [28] and the 2011 FITNESSGRAM® standards [27].

### Maturation status

Maturation status was assessed by the classification described by Tanner (self-reported pubertal status), which is based on the extent of hair covering of the pubertal region, (five stages: I-V) as: prepubescent (I-II), pubescent (III), and postpubescent (IV-V) [29] group. Each volunteer entered an isolated room, where, using a set of images exemplifying the various stages of sexual maturation, they categorized the development of their own genitalia (for boys), breasts (for girls), armpits (for boys) and pubic hair (for both genders). The reproducibility of our data reached 85%.

### Reliability health-related fitness measurements

All fitness measurements in a subsample of (*n =* 124) boys and (*n =* 105) girls [mean weight = 46.2 ± 12.4 kg, mean height = 1.50 ± 0.1 m, mean BMI = 19. 9 ± 3.1 kg/m^2^, mean age = 12.8 ± 2.4 years] were repeated by having the subject undergo the tests again 1 week later. The same inter-trial period has been used previously in similar reliability studies conducted with healthy young individuals [30]. In all the tests, we found almost excellent test-retest reliability [body mass (intraclass correlation, ICC = 0.983), BMI (ICC = 0.973), triceps SS (ICC = 0.864), subscapular SS (ICC = 0.859), %BF (ICC 0.897), maturation status (ICC = 0.856), and *course-navette* shuttle run test (ICC = 0.967)].

### Ethics statement

The study protocol was explained verbally to the participants and their parents/guardians before they gave their written consent. Participation in the study was fully voluntary and anonymous, with no explicit incentives provided for participation. This protocol was in accordance with the latest revision of the Declaration of Helsinki and was approved by the Review Committee for Research with Human Subjects at the University of Manuela Beltrán (Resolution UMB N° 02-1902-2014).

### Statistical analyses

The anthropometric characteristics of the study sample are presented as means, standard deviations (SD) or relative frequencies (n, %). Normality of the selected variables was verified using histograms and Q-Q plots. An independent *t-test* or *chi square* was employed to determine the differences in the participants’ anthropometric characteristics and aerobic capacity measurements between boys and girls. Multiple regression analysis was performed to determine the strongest morphological component predictor for cardiorespiratory fitness, with controlling of factors – age, gender, and Tanner staging. Statistics were calculated on SPSS V. 21 software for Windows (SPSS, Chicago, IL, USA), and the significance level was set at 5%.

## Results

The characteristics for the two components of the FUPRECOL health-related fitness test according to the sex and age of the study sample are shown in Table 1. The mean and standard deviation (±) values were as follows: age 14.3 ± 2.2 years, weight 46.1 ± 10.6 kg, height 148.2 ± 11.6 m, BMI 20.7 ± 2.7 kg/m^2^, waist circumference 70.0 ± 7.3 cm, subscapular skinfold 10.3 ± 3.7 mm, triceps skinfold 12.5 ± 6.1 mm and BF% 21.8 ± 5.5%. The prevalence of overweight and obesity were significantly higher in girls (*p =* 0.001). Girls had a significantly higher BF% and a significantly higher WC (*p =* 0.001). VO_2_max were significantly different between sexes, and boys had significantly higher scores in the cardiorespiratory component (*p =* 0.001). The proportion of subjects with an aerobic capacity indicative of future cardiovascular risk was 7.3%. An unhealthy aerobic capacity was observed in 3.8% of boys and 11.7% of girls (X^2^ *p =* 0.001).

**Table 1:** Characteristics of schoolchildren and adolescents [mean (SD) or frequencies], by sex

	Total (*n =* 576)	Boys (*n =* 319)	Girls (*n =* 257)	*P-*value
Morphologic component				
Age (years)	14.3 ± 2.2	14.4 ± 2.2	14.1 ± 2.2	0.198
Weight (kg)	46.1 ± 10.6	46.3 ± 11.3	45.8 ± 9.7	0.634
Height (m)	148.2 ± 11.6	150.4 ± 13.1	145.4 ± 8.8	0.000
Body mass index (kg/m^2^)	20.7 ± 2.7	20.1 ± 2.2	21.5 ± 3.1	0.001
Body mass index (z-score)	0.13 ± 0.54	−0.02 ± 0.87	0.44 ± 0.72	0.001
Waist circumference (cm)	70.0 ± 7.3	69.1 ± 6.6	71.1 ± 8.0	0.001
Subscapular skinfold (mm)	10.3 ± 3.7	8.3 ± 2.6	12.8 ± 3.3	0.001
Triceps skinfold (mm)	12.5 ± 6.1	9.2 ± 3.2	16.6 ± 6.5	0.001
BF (%)	21.8 ± 5.5	19.8 ± 4.5	24.2 ± 5.7	0.001
Weight status n (%)^a^				
Normal weight (≥ P5 to < P85)	494 (85.8)	302 (94.7)	192 (74.7)	0.001
Overweight (≥ P85 to < P95)	77 (13.4)	15 (4.7)	62 (24.1)	0.001
Obese (≥ P95)	5 (0.9)	2 (0.6)	3 (1.2)	0.001
Maturation status (%)				
Prepubescent/Pubescent/Postpubescent	30.6/35.7/33.7	34.6/30.3/35.1	34.8/30.0/ 35.1	0.309
Cardiorespiratory component				
20-m shuttle run (stage)	7.2 ± 2.5	8.6 ± 2.2	5.5 ± 1.8	0.001
VO_2_max (ml^*a*^kg^*a*^min^−1^)	47.7 ± 6.7	51.5 ± 4.7	43.1 ± 5.9	0.001
Future cardiovascular risk n (%)	42 (7.3)	12 (3.8)	30 (11.7)	0.001

Mean ± standard deviation, except weight status and future cardiovascular risk (%). Total: sample of boys and girls together. Differences between boys and girls calculated using one-way analysis of variance and weight status, tanner stage or future cardiovascular risk by X^2^ test. Body fat percentage (BF%)

^a^ Weight status was assessed by following the IOFT criteria, according to which individuals with a BMI below the 5th percentile and below the 85th percentile are well-nourished (normal weight); individuals at or above the 85th percentile are overweight; and those at or above the 95th percentile are obese [23]

Table 2 shows that adolescents (both boys and girls) with a healthy aerobic capacity have a lower morphological component (weight, BMI, WC, subscapular and triceps skinfold).

**Table 2:** Differences in the morphological components between healthy aerobic capacity and unhealthy aerobic capacity groups in Nasa Indian Community from Cauca, Colombia

Fitness component	Group	Aerobic capacity		*P-*value
		Unhealthy	Healthy	
Morphological component				
Weight (kg)	Total	51.9 ± 9.3	44.9 ± 10.4	<0.001
	Boys	60.3 ± 15.4	45.8 ± 10.9	<0.001
	Girls	51.0 ± 8.0	43.2 ± 9.4	<0.001
Body mass index (kg/m^2^)	Total	23.1 ± 3.0	20.2 ± 2.3	<0.001
	Boys	23.0 ± 3.6	20.0 ± 2.0	<0.001
	Girls	23.1 ± 3.0	20.6 ± 2.8	<0.001
Waist circumference (cm)	Total	76.0 ± 7.1	68.8 ± 6.7	<0.001
	Boys	78.6 ± 8.8	68.7 ± 6.3	<0.001
	Girls	75.7 ± 6.9	68.8 ± 7.4	<0.001
Subscapular skinfold (mm)	Total	18.8 ± 5.8	11.2 ± 5.3	<0.001
	Boys	12.0 ± 2.9	9.1 ± 3.1	0.006
	Girls	19.5 ± 5.6	15.0 ± 6.3	<0.001
Triceps skinfold (mm)	Total	13.6 ± 3.7	9.6 ± 3.2	<0.001
	Boys	10.8 ± 5.4	8.2 ± 2.4	0.002
	Girls	13.9 ± 3.4	12.1 ± 3.1	<0.001
Body fat (%)	Total	26.7 ± 4.6	20.8 ± 5.1	<0.001
	Boys	24.6 ± 6.0	19.7 ± 4.3	<0.001
	Girls	26.9 ± 4.4	22.7 ± 5.7	<0.001

Mean ± standard deviation. Total: sample of boys and girls together. Differences between total, boys and girls calculated using one-way analysis of variance. Group and aerobic capacity level (healthy and unhealthy) as per the FITNESSGRAM reference criteria [25]

Multiple regression analysis in Table 3 showed that weight (ß = −0.206, *p <* 0.001), BMI (ß = −0.124, *p <* 0.001), WC (ß = −0.227, *p <* 0.001) and body fat percentage (ß = −0.249, *p <* 0.001) were significant predictor for CRF levels.

**Table 3:** Standardised coefficient (ß) between morphologic component and cardiorespiratory fitness levels, using multiple regression analysis^a^

Morphologic component	Cardiorespiratory fitness	*P-*value
	ß	
Weight (kg)	−0.206	<0.001
Body mass index (kg/m^2^)	−0.124	<0.001
Waist circumference (cm)	−0.227	<0.001
BF (%)	−0.249	<0.001

^a^Control for age, gender, and sexual maturity

**Fig. 1:**
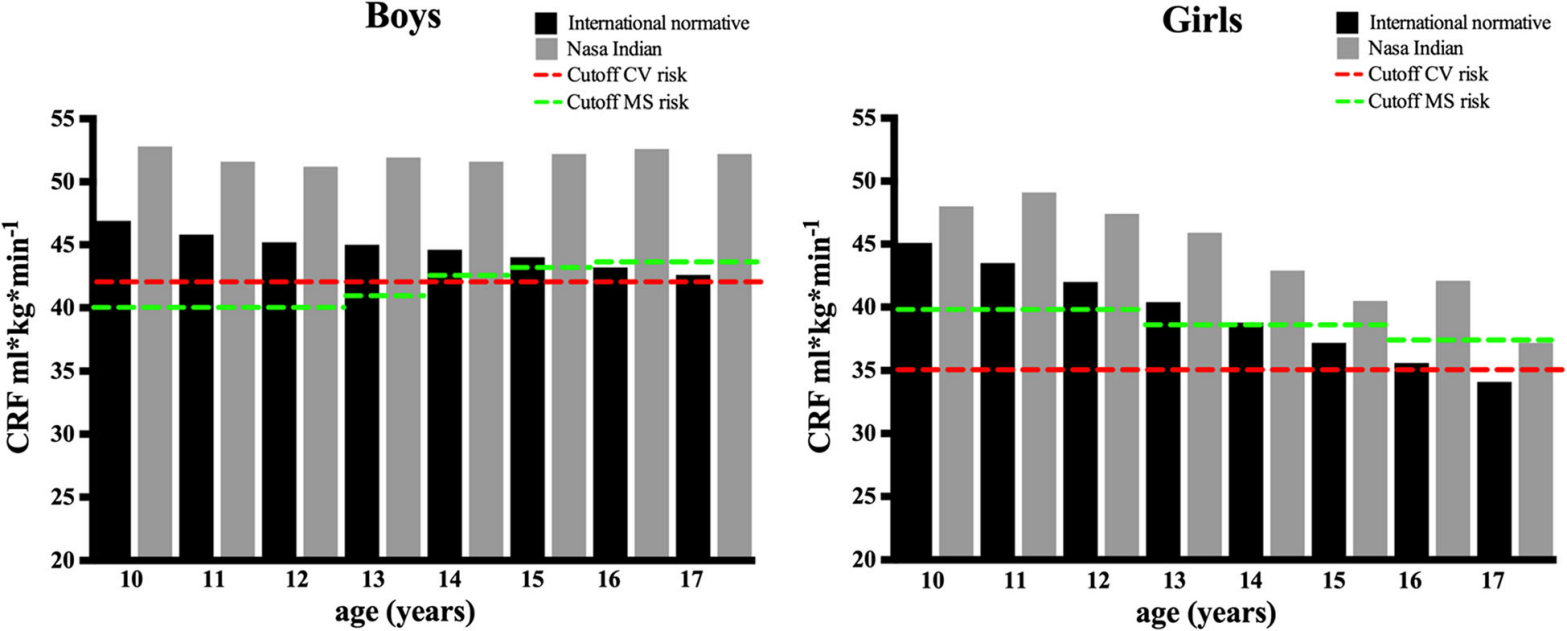
CRF comparison between Nasa Indian and an international normative 20 m shuttle run test considering a cutoff to avoid cardiovascular risk and metabolic syndrome in children and adolescents. CV: cardiovascular, MS: Metabolic syndrome, CRF: Cardiovascular Fitness

Figure 1 shows a comparison between Nasa Indian and a 50-country CRF average (high and middle income) [30] and considering an age-and-sex-specific cut-off point to avoid cardiovascular [31] and metabolic syndrome risk [27 in children and adolescents. Overall, it is appreciated that both boys and girls Nasa Indian have a high level of CRF than a considerable sample of children from Africa, Asia, Europe, Latin American and The Caribbean, Northern American, and Oceania countries. Nasa Indian boys’ CRF is maintained above 50 ml*kg*min^−1^ across 10 to 17 years old, while in girls CRF appears to be lower as the age advances.

## Discussion

The main finding of this study was the proportion of subjects with a CRF indicative of future cardiovascular risk was 7.3%. By sex, 3.8% of boys and 11.7% of girls (X^2^ = 0.001) displayed an unhealthy CRF. This study shows the first published research study using the FUPRECOL test battery in a sample of Colombian indigenous adolescents. The main strength of this study, and in terms of how CRF is indicative of future cardiovascular risk, is the strict standardization of the fieldwork among the Indian community.

In 2009, Ruiz et al. [10] systematically reviewed whether CRF in childhood was a predictor of health independent of body fatness parameters or weight status later in life.

On the other hand, the results obtained in this study show that, in general, children and adolescents with a healthy CRF had a better morphological outcome (Table 2). In addition, they had lower waist circumference and body fat values, which are all factors related to an improved cardiovascular profile [32]. Compared with previous studies, this difference could be a result of international variation in adiposity patterns or because the health-related CRF cut-points used in this study may have discriminated adiposity better than the selected Healthy Fitness Zone standards. This evidence demonstrates that physical education teachers play the role of health promoters for their students. Therefore, the school setting is exceptional for promoting exercise habits and preserving cardiovascular health [33].

Other studies have investigated the influence of fitness on adiposity indices using multiple linear regression analysis [34, 35]. Body fat percentage and WC were the strongest predictor to cardiorespiratory fitness levels in our findings after adjusting factors of age, gender and sexual maturity. Ara et al. [36]. revealed cardiorespiratory fitness was the strongest predictor of BMI, body fat and subcutaneous truncal fat, as compared to physical activity level. Another study showed that cardiorespiratory fitness, was inversely associated with both BMI and WC after controlling factors of age, sexual maturity and active commuting to school [37]. A third study in Malaysian adolescent revealed an inverse association between waist circumference and fitness score among apparently healthy adolescents. This reiterates the importance of body composition as a measurement tool of health status for children and adolescent. It is a strong predictor for cardio-metabolic risk factors in children and adolescent [3, 6, 31]. Previosly studies found a significant association between childhood adiposity and unfavorable metabolic profiles [38, 39]. A probable hypotheses way is the release of free fatty acids through lipolysis of omentum and mesenteric adipocytes, which in turn triggers vascular fuction, insulin resistance and dyslipidemia [40, 41]. All these warrant the importance of active intervention and healthy lifestyle to start at a indigenous population.

In this study they found strong evidence indicating that lower level of CRF in childhood and adolescence was associated to cardiovascular diseases risk factors, arterial stiffness, and metabolic syndrome in adulthood. Recently, the same authors have identified as “pathological fitness level” a CRF below to 25^th^ percentile in youth [42]. And in 2016, they published the cut points of CRF to avoid cardiovascular disease risk in children and adolescents (used in our Fig. 1) [31]. This powerful evidence demonstrates on the one hand, the importance of CRF as a health marker in children and adolescents and its implications on health in adulthood, and on the other, it provides us CRF cut-offs to establish a “warning sign” to detect the population at risk early.

Despite the above, few studies available in the literature have investigated poor physical fitness profiles with similar socio-cultural characteristics and from the same ethnic origin [5, 32, 43]. In this study, the proportion of children and adolescents with a CRF indicative of future cardiovascular disease (CVD) was 7.3%. By sex, the proportion of subjects with an unhealthy CRF were 3.8% in boys and 11.7% in girls (X^2^ = 0.001). These results are substantially lower than those observed in previous international studies conducted with adolescents (26% of boys and 55% of girls in Chile [44]; 38% of boys and 43% of girls in the European Union [45]; 29% of boys and 23% of girls in Australia [46]; 29% of boys and 31% of girls in the United States [26]; and 11.5% of children and 49.1% of adolescents in Argentina [47].

Previous research indicates that low fitness levels can linger on into adulthood in which low CRF is associated with increased mortality risk [33, 48, 49]. However, differences in the environment alone do not appear to tell the entire story, particularly in elucidating why certain populations and ethnic groups experience a disproportionately high prevalence of CVD when they adopt a western lifestyle [5]. The classic example of this is the Pima Indians, who when living a traditional rural lifestyle in Mexico are lean, active and have a low diabetes prevalence, but when living in the US, are generally obese and have prevalence of diabetes mellitus and other non-communicable diseases in ~40% of the adult population [50]. This pattern is also evident in other indigenous populations throughout the Americas [51] and Australia [52]. Future studies will need to confirm the extent to which this phenotype remains at cardio-metabolic disease risk compared with counterparts with healthy CRF.

Compared to Nasa Indian adolescents in another studies, our sample saw a better performance on cardiorespiratory components [46, 47, 53, 54]. Figure 1 shows a CRF comparison between Nasa Indians and an international normative 20-m shuttle run test from 50 countries [30]. We have considering two cutoffs, the first (red line), to avoid cardiovascular risk recently published by Ruiz et al. in 2016 (42 ml*kg*min^−1^ for boys and 35 ml*kg*min^−1^ for girls) [31], and the second (green line), which is linked to metabolic syndrome from an cross-sectional study conducted by Welk et al., in 2011 (40–44 ml*kg*min^−1^ for boys and 38–40 ml*kg*min^−1^ for girls) [27].

It can be seen that Nasa Indian boys exceed both cutoffs (cardiovascular and metabolic syndrome risk) from 10 to 17 years old, and their CRF values are higher than the sample of 50 countries boys from the international study. In contrast, the boys’ CRF from 50 countries decreased, and at the age of 17 they are slightly above the cardiovascular risk cutoff (0.6 ml*kg*min^−1^). In girls, the Nasa Indian sample exceeds both cutoffs until the age of 16, whereas the girls’ CRF from the international normative 20-m shuttle run test is only until 14 years old.

Explaining the differences between urban and rural adolescents remains speculative. One possible explanation for the differences in physical fitness profiles among indigenous adolescents may be the differences in moderate to vigorous physical activity [55] or differences in body fat distribution [56]. It is possible the FITNESSGRAM CRF cut-points, which were developed to discriminate body fatness parameter, identified enough excess adiposity among Nasa Indian sample. This observation could also be a result of the selected cut-points for CRF, reinforcing the need for development of aerobic capacity health-related standards to discriminate cardio-metabolic health in youth [56]. Nevertheless, in Latin America, urban Ecuadorian adolescents had better physical fitness and blood lipid profiles than rural adolescents, independent of sedentary time [56–58]. However, other environmental and socio-economic correlates must be explored.

There are some limitations on this study. Firstly, due to its cross-sectional nature we cannot discern the direction of the observed associations between CRF and future cardiovascular risk, which may indeed be reciprocal [59, 60]. Secondly, we did not measure important variables associated with cardiovascular disease such as blood lipids, sex hormone levels, physical activity or familial health background. Third, the estimation of VO_2_max from the FITNESSGRAM standards of the 20-m shuttle run is known to vary with the equation used. Our decision to categorize VO_2_max fitness according to health predictive value instead of using continuous variables can be considered a limitation of the study. Another potential limitation is the equation used to estimate VO_2_max, which may underestimate cardiorespiratory fitness by up to 12% relative to other methods and therefore may, in isolation, have inflated the prevalence of unhealthy aerobic capacity [60]. However, such limitations do not compromise the results obtained when validating these results.

Finally, the small number of studies on the indigenous population did not allow us to make comparisons with the results of this work. Furthermore, despite their larger burden of chronic disease and the alarming increase in the prevalence of obesity in children and adolescents in Latin America [61], lower middle-income countries such as Kenya are also substantially underrepresented in physical activity intervention research [62]. The discordant fitness-body fatness parameters pairs highlighted in this study bolster the argument for the inclusion of CRF assessment or data interpretation for youth in clinical practice [63]. On the other hand, our decision to categorize CRF according to health predictive value instead of using continuous variables can be considered a strength of the study as it allowed for greater public health interpretability. CRF has been suggested before when estimating metabolic health risks associated with obesity or physical inactivity. Another potential strength of the study was the use of health-related, valid, and reliable field tests recommended for Latin-American youth fitness assessment [64].

## Conclusions

In summary, our results show for the first time that Colombian Nasa Indian children and adolescents aged 10–17.9 years have a low rate of unhealthy CRF, which significantly reduces their future cardiovascular risk. Although the known loss of CRF is a serious consequence of the future risk of non-communicable diseases, the deterioration of aerobic capacity deserves increased attention among indigenous population.

## Abbreviations

BF%: Percentage body fat
BMI: Body mass index
CPAFLA: The Canadian Physical Activity, Fitness and Lifestyle Approach
CRF: Cardiorespiratory fitness
CVD: Cardiovascular disease
EUROFIT: The European Phisical Fitness
FUPRECOL: in Spanish, Asociación de la Fuerza Prensil con Manifestaciones de Riesgo cardiovascular Tempranas en Niños y Adolescentes Colombianos)
ICC: Intraclass correlation
IOTF: International Obesity Task Force
PYFP: The Presidential Youth Fitness Program
SD: Standard deviations
SS: Skinfold thicknesses
TEM: Technical error of measurement
VO_2_max: Maximum volume of oxygen
WC: Waist circumference

## References

[1] Gualteros JA, Torres JA, Umbarila-Espinosa LM, Rodríguez-Valero FJ, Ramírez-Vélez R (2015). A lower cardiorespiratory fitness is associated to an unhealthy status among children and adolescents from Bogotá, Colombia. Endocrinol Nutr.

[2] Kelishadi R, Gheiratmand R, Ardalan G, Adeli K, Mehdi Gouya M, CASPIAN Study Group (2007). Association of anthropometric indices with cardiovascular disease risk factors among children and adolescents: CASPIAN Study. Int J Cardiol.

[3] Ekelund U, Anderssen SA, Froberg K, Sardinha LB, Andersen LB, Brage S (2007). Independent associations of physical activity and cardiorespiratory fitness with metabolic risk factors in children: the European youth heart study. Diabetologia.

[4] Silva-Santos S, Santos A, Vale S, Mota J. Motor fitness and preschooler children obesity status. J Sports Sci. 2016;15:1–5. 10.1080/02640414.2016.1232486 27748155

[5] Ramos-Sepúlveda JA, Ramírez-Vélez R, Correa-Bautista JE, Izquierdo M, García-Hermoso A (2016). Physical fitness and anthropometric normative values among Colombian-Indian schoolchildren. BMC Public Health.

[6] Hurtig-Wennlof A, Ruiz JR, Harro M, Sjostrom M (2007). Cardiorespiratory fitness relates more strongly than physical activity to cardiovascular disease risk factors in healthy children and adolescents: the European Youth Heart Study. Eur J Cardiovasc Prev Rehabil.

[7] Ramírez-Vélez R, Tordecilla-Sanders A, Correa-Bautista JE, Peterson MD, Garcia-Hermoso A. Handgrip Strength and Ideal Cardiovascular Health among Colombian Children and Adolescents. J Pediatr. 2016;179:82-9. 10.1016/j.jpeds.2016.08.099 27720242

[8] Steene-Johannessen J, Anderssen SA, Kolle E, Andersen LB (2009). Low muscle fitness is associated with metabolic risk in youth. Med Sci Sports Exerc.

[9] Norman K, Stobaus N, Gonzalez MC, Schulzke J-D, Pirlich M (2010). Hand grip strength: outcome predictor and marker of nutritional status. Clin Nutr.

[10] Ruiz JR, Castro-Pinero J, Artero EG, Ortega FB, Sjostrom M, Suni J (2009). Predictive validity of health-related fitness in youth: a systematic review. Br J Sports Med.

[11] Cooper Institute for Aerobics Research (2004). The Prudential Fitnessgram: Test Administration Manual.

[12] The President’s Council on Physical Fitness and Sports. The President’s Challenge: The Health Fitness Test. Available at: https://www.fitness.gov/participate-in-programs/presidential-youth-fitnessprogram/. Accessed 4 Oct 2012.

[13] Council of Europe Committee for the Development of Spor (1993). EUROFIT: Handbook for the EUROFIT Tests of Physical Fitness.

[14] Canadian Society for Exercise Physiology (CSEP) (2003). The Canadian Physical Activity, Fitness & Lifestyle Approach (CPAFLA): CSEPHealth & Fitness Program’s Health-Related Appraisal and Counselling Strategy.

[15] Ramírez-Vélez R, Rodrigues-Bezerra D, Correa-Bautista JE, Izquierdo M, Lobelo F (2015). Reliability of Health-Related Physical Fitness Tests among Colombian Children and Adolescents: The FUPRECOL Study. PLoS One.

[16] Ramírez-Vélez R, Daza F, González-Jiménez E, Schmidt-RioValle J, González-Ruíz K, Correa-Bautista JE. Cardiorespiratory Fitness, Adiposity, and Cardiometabolic Risk Factors in Schoolchildren: The FUPRECOL Study. West J Nurs Res. 2016. [Epub ahead of print] 10.1177/0193945916664900 27550468

[17] Williams DR (1997). Race and health: basic questions, emerging directions. Ann Epidemiol.

[18] Caprio S, Daniels SR, Drewnowski A, Kaufman FR, Palinkas LA, Rosenbloom AL (2008). Influence of race, ethnicity, and culture on childhood obesity: implications for prevention and treatment. Obesity (Silver Spring).

[19] Godin K, Leatherdale ST, Elton-Marshall T (2015). A systematic review of the effectiveness of school-based obesity prevention programmes for First Nations, Inuit and Métis youth in Canada. Clin Obes.

[20] Ali MM, Rizzo JA, Amialchuk A, Heiland F (2014). Racial differences in the influence of female adolescents’ body size on dating and sex. Econ Hum Biol.

[21] Enes Romero P, Cano Gutiérrez B, Alvarez Gil N, Martín-Frías M, Alonso Blanco M, Barrio CR (2013). Ethnic influence on the prevalence of metabolic syndrome in an obese pediatric population. An Pediatr (Barc).

[22] Departamento Administrativo Nacional de Estadística (DANE) (2007). Los grupos étnicos de Colombia en el censo de 2005.

[23] Cole TJ, Flegal KM, Nicholls D, Jackson AA. Body mass index cut offs to define thinness in children and adolescents: international survey. BMJ. 2007;335. 10.1136/bmj.39238.399444.55 PMC1934447 17591624

[24] Slaughter MH, Lohman TG, Boileau RA, Horswill CA, Stillman RJ, Van Loan MD, et al. Skinfold equations for estimation of body fatness in children and youth. Hum Biol. 1988;60:709–23. 3224965

[25] Leger LA, Mercier D, Gadoury C, Lambert J (1988). The multistage 20 m shuttle run test for aerobic fitness. J Sports Sci.

[26] Ramírez-Vélez R, Palacios-López A, Humberto Prieto- D, Enrique Correa-Bautista J, Izquierdo M, Alonso-Martínez A, Lobelo F. Normative reference values for the 20 m shuttle-run test in a population-based sample of school-aged youth in Bogota, Colombia: the FUPRECOL study. Am J Hum Biol. 2017;29:e22902. 10.1002/ajhb.22902 PMC5298048 27500986

[27] Welk GJ, Laurson KR, Eisenmann JC, Cureton KJ (2011). Development of Youth Aerobic-Capacity Standards Using Receiver Operating Characteristic Curves. Am J Prev Med.

[28] Lobelo F, Pate RR, Dowda M, Liese AD, Ruiz JR (2009). Validity of cardiorespiratory fitness criterion-referenced standards for adolescents. Med Sci Sports Exerc.

[29] Matsudo SMM, Matsudo VKR (1994). Self-assessment and physician assessment of sexual-maturation in Brazilian boys and girls – concordance and reproducibility. Am J Hum Biol.

[30] Tomkinson GR, Lang JJ, Tremblay MS, Dale M, LeBlanc AG, Belanger K, Ortega FB, Léger L. International normative 20 m shuttle run values from 1 142 026 children and youth representing 50 countries. Br J Sports Med. 2016. doi: 10.1136/bjsports-2016-095987. [Epub ahead of print] 10.1136/bjsports-2016-095987 27208067

[31] Ruiz JR, Cavero-Redondo I, Ortega FB, Welk GJ, Andersen LB, Martinez-Vizcaino V. Cardiorespiratory fitness cut points to avoid cardiovascular disease risk in children and adolescents; what level of fitness should raise a red flag? A systematic review and meta-analysis. Br J Sports Med. 2016. doi: 10.1136/bjsports-2015-095903. [Epub ahead of print] 10.1136/bjsports-2015-095903 27670254

[32] Ortega FB, Ruiz JR, Castillo MJ, Moreno LA, González-Gross M, Wärnberg J (2005). Low level of physical fitness in Spanish adolescents. Relevance for future cardiovascular health (AVENA study). Rev Esp Cardiol.

[33] Suriano K, Curran J, Byrne SM, Jones TW, Davis EA (2010). Fatness, fitness, and increased cardiovascular risk in young children. J Pediatr.

[34] Hussey J, Bell C, Bennett K, O’Dwyer J, Gormley J (2007). Relationship between the intensity of physical activity, inactivity, cardiorespiratory fitness and body composition in 7-10-year-old Dublin children. Br J Sports Med.

[35] Hanifah RA, Majid HA, Jalaludin MY, Al-Sadat N, Murray LJ, Cantwell M, Su TT, Nahar AM (2014). Fitness level and body composition indices: cross-sectional study among Malaysian adolescent. BMC Public Health.

[36] Ara I, Moreno LA, Leiva MT, Gutin B, Casajus JA (2007). Adiposity, physical activity, and physical fitness among children from Aragon, Spain. Obesity.

[37] Ortega FB, Tresaco B, Ruiz JR, Moreno LA, Martin-Matillas M, Mesa JL, Warnberg J, Bueno M, Tercedor P, Gutierrez A (2007). Cardiorespiratory fitness and sedentary activities are associated with adiposity in adolescents. Obesity.

[38] Wang PG, Gong J, Wang SQ, Talbott EO, Zhang B, He QQ (2011). Relationship of body fat and cardiorespiratory fitness with cardiovascular risk in Chinese children. PLoS One.

[39] Reuter CP, da Silva PT, Renner JD, de Mello ED, Valim AR, Pasa L, da Silva R, Burgos MS (2016). Dyslipidemia is Associated with Unfit and Overweight-Obese Children and Adolescents. Arq Bras Cardiol.

[40] Berenson GS, Srinivasan SR, Xu JH, Chen W (2016). Adiposity and Cardiovascular Risk Factor Variables in Childhood Are Associated With Premature Death From Coronary Heart Disease in Adults: The Bogalusa Heart Study. Am J Med Sci.

[41] Freedman DS, Ogden CL, Kit BK (2015). Interrelationships between BMI, skinfold thicknesses, percent body fat, and cardiovascular disease risk factors among U.S. children and adolescents. BMC Pediatr.

[42] Ruiz JR, Huybrechts I, Cuenca-García M, Artero EG, Labayen I. Meirhaeghe A, on behalf of the HELENA study group Cardiorespiratory fitness and ideal cardiovascular health in European adolescents. Heart. 2015;101:766–73. 10.1136/heartjnl-2014-306750 25489050

[43] Gill JM, Bhopal R, Douglas A, Wallia S, Bhopal R (2011). Sitting Time and Waist Circumference Are Associated With Glycemia in U.K. South Asians: Data from 1,228 adults screened for the PODOSA trial. Diabetes Care.

[44] Celis-Morales CA, Perez-Bravo F, Ibañes L, Sanzana R, Hormazabal E, Ulloa N (2011). Insulin resistance in Chileans of European and indigenous descent: evidence for an ethnicity X environment interaction. PLoS One.

[45] Minatto G, Petroski EL, Silva DA (2013). Body fat, muscular and cardiorespiratory fitness according to sexual maturation among Brazilian adolescents from a town of German colonization. Rev Paul Pediatr.

[46] Tremblay MS, Shields M, Laviolette M, Craig CL, Janssen I (2010). Connor Gorber S Fitness of Canadian children and youth: results from the 2007–2009 Canadian Health Measures Survey. Health Rep.

[47] Secchi JD, García GC, España-Romero V, Castro-Piñero J (2014). Physical fitness and future cardiovascular risk in argentine children and adolescents: an introduction to theALPHA test battery. Arch Argent Pediatr.

[48] Blair SN, Kohl HW, Paffenbarger RS, Clark DG, Cooper KH, Gibbons LW (1989). Physical fitness and all-cause mortality. A prospective study of healthy men and women. JAMA.

[49] Schulz LO, Bennett PH, Ravussin E, Kidd JR, Kidd KK, Esparza J (2006). Effects of traditional and western environments on prevalence of type 2 diabetes in Pima Indians in Mexico and the U.S. Diabetes Care.

[50] Yu CH, Zinman B (2007). Type 2 diabetes and impaired glucose tolerance in aboriginal populations: a global perspective. Diabetes Res Clin Pract.

[51] Eisenmann JC (2007). Aerobic fitness, fatness and the metabolic syndrome in children and adolescents. Acta Paediatr.

[52] Ekelund U, Luan J, Sherar LB, Esliger DW, Griew P, Cooper A (2012). Moderate to vigorous physical activity and sedentary time and cardiometabolic risk factors in children and adolescents. JAMA.

[53] Garber MD, Sajuria M, Lobelo F (2014). Geographical variation in health-related physical fitness and body composition among Chilean 8th graders: a nationally representative cross-sectional study. PLoS One.

[54] Ortega FB, Artero EG, Ruiz JR, Espana-Romero V, Jimenez-Pavon D, Vicente-Rodriguez G (2011). Physical fitness levels among European adolescents: the HELENA study. Br J Sports Med.

[55] Ried-Larsen M, Grøntved A, Møller NC, Larsen KT, Froberg K, Andersen LB (2014). Associations between objectively measured physical activity intensity in childhood and measures of subclinical cardiovascular disease in adolescence: prospective observations from the European Youth Heart Study. Br J Sports Med.

[56] Andrade S, Ochoa-Avilés A, Lachat C, Escobar P, Verstraeten R, Van Camp J (2014). Physical fitness among urban and rural Ecuadorian adolescents and its association with blood lipids: a cross-sectional study. BMC Pediatr.

[57] Ramírez-Vélez R, Meneses-Echavez JF, González-Ruíz K, Correa JE (2014). Muscular fitness and cardiometabolic risk factors among Colombian young adults. Nutr Hosp.

[58] Boiarskaia EA, Boscolo MS, Zhu W, Mahar MT (2011). Cross-validation of an equating method linking aerobic FITNESSGRAM(R) field tests. Am J Prev Med.

[59] Pate RR, Wang CY, Dowda M, Farrell SW, O’Neill JR (2006). Cardiorespiratory fitness levels among US youth 12 to 19 years of age: findings from the 1999–2002 National Health and Nutrition Examination Survey. Arch Pediatr Adolesc Med.

[60] Carrel AL, Bowser J, White D, Moberg DP, Weaver B, Hisgen J (2012). Standardized childhood fitness percentiles derived from school-based testing. J Pediatr.

[61] Rivera JÁ, de Cossío TG, Pedraza LS, Aburto TC, Sánchez TG, Martorell R (2014). Childhood and adolescent overweight and obesity in Latin America: a systematic review. Lancet Diabetes Endocrinol.

[62] Muthuri SK, Wachira LJ, Onywera VO, Tremblay MS (2014). Correlates of objectively measured overweight/obesity and physical activity in Kenyan school children: results from ISCOLE-Kenya. BMC Public Health.

[63] Sarmiento OL, Parra DC, González SA, González-Casanova I, Forero AY, Garcia J (2014). The dual burden of malnutrition in Colombia. Am J Clin Nutr.

[64] González SA, Castiblanco MA, Arias-Gómez LF, Martinez-Ospina A, Cohen DD, Holguin GA, et al. Results From Colombia's 2016 Report Card on Physical Activity for Children and Youth. J Phys Act Health. 2016;13:S129-36. 10.1123/jpah.2016-0369 27848732

